# Working toward a transdisciplinary approach to teaching and learning planetary health–A collective reflection

**DOI:** 10.3389/fpubh.2022.1039736

**Published:** 2022-12-05

**Authors:** Cato Dambre, Julia Gabriela Strack Diaz, Rana Orhan, Doreen Montag, Indira van der Zande, Valentina Gallo

**Affiliations:** ^1^Faculty of Medicine and Health Sciences, Ghent University, Ghent, Belgium; ^2^Department of Sustainable Health, Campus Fryslân, University of Groningen, Leeuwarden, Netherlands; ^3^University College Fryslan, Campus Fryslân, University of Groningen, Leeuwarden, Netherlands; ^4^The Association of Schools of Public Health in the European Region, Brussels, Belgium; ^5^Unit for Global Public Health, Wolfson Institute of Population Health, Queen Mary University of London, London, United Kingdom

**Keywords:** transdisciplinarity, innovative teaching, planetary health, thematic analysis, focus group, pedagogy, transcultural approach

## Abstract

**Background:**

In order to educate the next generation of leaders to work at reverting the damaging effects of the Anthropocene, there is an increasing need to incorporate more environmental-related aspects in all teaching programmes, including the health-related. Planetary health is a complex field which can benefit from a transdisciplinary pedagogical approach. The aim of this research was to evaluate an approach working toward transdisciplinarity applied to a course of Planetary Health taught at the Bachelor degree Global Responsibility & Leadership of the University of Groningen through substantive feedback and reflections from the students.

**Methods:**

By the end of the course, a focus group was conducted with the students inviting them to reflect on the different aspects of the pedagogical approach, evaluating their effectiveness. A thematic analysis was conducted on the transcribed focus group.

**Results:**

The students appreciated the added value of working toward a transdisciplinary approach and peer-to-peer learning and teaching adopted in the Planetary Health course, as a way of enhancing their learning experience. They pointed out the need of incorporating a transcultural approach into the transdisciplinary one, as a way not only to improve their learning experience, but also to enrich the transdisciplinarity itself.

**Conclusion:**

Incorporating a process toward transdisciplinary and transcultural teaching of planetary health into undergraduate programmes was found to be of added value. The peer-to-peer horizontal learning opportunities were seen as a way for taking advantage of the collaborative, informal teaching and community building serving the overall scope of the course.

## Introduction

Our planet is rapidly changing. We have entered a new era, the Anthropocene, characterized by the impact of human activities on the planet and every living species on it (1). Anthropogenic activities and their consequences such as climate change, biodiversity loss, land use change, and pollution threaten human health directly and indirectly (2). They have an impact on the natural environment we depend on (e.g., air quality, arable land, temperature), and they are directly responsible for the increase of mental health, infectious diseases, non-communicable diseases, physical trauma, displacement and malnutrition, all major global public health concerns (1, 2).

In order to revert the trends and limit the damages caused during the Anthropocene, strong international political will is needed; however, economic interests often compete with environmental stances (3). For sustainable changes in the long term, the new generation, already very sensitive to the topic (4), needs to be educated to evaluate, understand, and ponder the intricate net of consequences that human activity has on the health of humans and the planet. Concepts like One Health and Ecohealth were developed, focussing on the interaction between animal, human and ecosystem health (5). Simultaneously, the field of Global Health emerged, concerned with improving health and achieving health equity for all people worldwide (6). Following these concepts and building upon the inter- and transdisciplinary work that has been done in those disciplines, a new concept was developed that combined them all: Planetary Health. Planetary health is defined as “the health of human civilization and the state of the natural systems on which it depends” (7), bit is also referred to as a “solution-oriented, transdisciplinary field and a social movement focused on analyzing and addressing the impacts of human disruptions to Earth's natural systems on human health and all life on Earth” (8).

In comparison to other approaches related to environmental health, planetary health is not a fully developed concept, but it has been gaining traction due to its introduction of the importance of sustainability, as well as inclusion of factors such as gender and socioeconomic background (5). Because of its importance and its comprehensive approach, interest in planetary health education has been increasing across different disciplines, institutions, and world areas (1, 9). Importantly, a distinctive factor of the study of planetary health, is the complexity of the problems it aims to investigate. The investigation of such complexities relies on the expertise coming from several different disciplines to unpack, understand, and analyse individual problems and their extended network of interaction. This naturally requires going beyond the disciplinary approach, and instead implementing new, more permeable approaches suitable for this field complexity.

Throughout this paper we refer to the concepts of *multidisciplinarity* as the approach that draws on knowledge from different disciplines, allowing knowledge to remain within their boundaries; *interdisciplinarity* as the process of analyzing, synthesizing and harmonizing links between disciplines into a coordinated and coherent whole; and *transdisciplinarity* as the ultimate integration of the natural, social and health sciences in a humanities context, allowing (academic and non-academic) disciplines to transcend their traditional boundaries (10). Aware of the existence of multiple definitions, these were chosen as developed in an evidence-based manner from health and education studies (10).

It was previously suggested that transdisciplinary in teaching and learning is a key pedagogical approach for specific fields characterized by intrinsic complexity and at the intersection of traditional disciplinary fields (11–13). Planetary health would be perfectly suited to be studied with a transdisciplinary approach. This in fact allows transcending disciplinary boundaries and creating a major reconfiguration of disciplinary divisions within a systemic, global and integrated perspective (14, 15). This approach is particularly suited to address contemporary challenges and includes the idea of extended cross-discipline peer-review. Ideally, stakeholders from outside the academic field would also contribute to the construction of knowledge and co-create, together with scientists, practical solutions to social problems (16, 17). Even though the stakeholder aspect was not specifically included in the present approach, it remains transdisciplinary in essence as it starts from the complexity unpacking it into simpler issues, and adds the cultural dimension to the pure encounter of disciplines. To what extent an academic approach working toward transdisciplinarity in Planetary health is suited for any level of teaching, and how it is enriched by classes of students coming from different backgrounds remain important topics to be studied.

As such, this research aims at evaluating an academic approach working toward transdisciplinarity to teaching and learning Planetary health by applying it to the case study of a newly developed Planetary Health course within the BSc Global Responsibility & Leadership at University of Groningen and inviting the students to collectively reflect on its merit.

## Methods

### BSc global responsibility and leadership

The Global Responsibility & Leadership (GRL) bachelor program at University of Groningen, is an interdisciplinary and international bachelor whose curricula is founded on the sustainable development goals (SDG's), and modeled following the liberal arts and sciences philosophy, featuring the main motto “global challenges, local solutions.” As such, the programme offers a broad as well as in-depth academic training, with a strong focus on social responsibility and (personal) leadership (18). The three-year undergraduate degree is structured into a series of mandatory introductory courses of a selection of six different areas of scholarship (traditionally described as disciplines) and an extensive skill-based training in year one, forming the shared academic core, or foundation year. In the second and third years, student follow elective courses which are divided into three main majors that students can choose from: Responsible Humanity, Responsible Governance and Responsible Planet. The teaching and learning environment is fundamentally learned focused: classes are small-scale with typically up to a maximum of 25 students per class, and teacher act as coaches who facilitate discussion and critical thinking.

### The planetary health course

The planetary health course is a 9-week elective module designed as an overarching course open to third year students from any track and belonging to any major. As students in this course have already completed 2 years of study, they have started building slightly different academic backgrounds. This allowed subdividing the students by background in order to prompt a disciplinary encounter of peer-to-peer learning.

The course has been designed using an approach working toward transdisciplinarity. It focused on interconnectedness of issues, and how these generate intricate systems to be analyzed in all their complexity. The learning outcomes of the course were:

1. Describe the main determinants of the complex interaction between human and planetary health.
2. Examine various theoretical concepts in light of their application to planetary health determinants.
3. Apply an inter/transdisciplinary approach in practice by fruitfully interacting with expert from different fields.
4. Generate an evidence-based analysis of a complex issue in planetary health applying the principles of system dynamics.
5. Reflect on the deep meaning of the connection between humans and nature.
6. Present a planetary health-related seminar with an interdisciplinary team to a wider academic audience, and discuss its content.

The taught component of the module was structured around six abstract concepts (equilibrium, scarcity, common good, tipping point, belonging and risk), which set the main themes of each week. These served as a starting point for multiple disciplinary reflections on planetary health.

The course also combined vertical and horizontal learning. Vertical teaching included expanding on each one of the abstract concepts with two lectures given by two experts from two different disciplines; for example the topic of “belonging” included one lecture entitled “Acceptance, belonging, and Agency,” an overview of social science research on migration, with particular emphasis on solastalgia (19), and another entitled “The epigenetic Landscape” tackling the concept of belonging from a biomedical perspective exploring the environmental hallmark on the human genome (20). Horizontal teaching was promoted with student-led sessions. Twice during the course, students with a specific background (social, environmental, or governance) were asked to deepen their knowledge on a topic, and plan as well as lead an entire session teaching that topic to their peers. This included circulating any preparation material, and conducting any formative assessment, if needed.

This course was designed to transcend the academic boundaries of individual disciplines creating a shared knowledge and understanding of reality, generating space for deeper reflection. For example, it presented students with complex problem to unpack in all their complexity working on the interactions of individual disciplinary issues. Further, students and teachers were explicitly prompted to transcend their disciplinary fields entering a no-man-land of dialogue and potential cross-fertilization. Finally the teaching space opened the door to broader reflections on the topic addressed, from cultural meanings to activism. The course, however, did not yet managed to meet all the standards of what is commonly defined as transdisciplinarity, as it did not include any societal stakeholders into its current structure. For this reason in this paper we describe this approach as working toward transdisciplinarity.

The course was run for the first time from November 2021 to January 2022, a total of eight students participated, four from Responsible Governance, three from Responsible Humanity, and one from Responsible Planet (Table 1).

**Table 1:** Participants' characteristics (*N* = number of participants).

		***N***
Background (tracks)	Humanities	3
	Governance	4
	Environment	1
Sex	Male	2
	Female	6
Nationality	Western European	6
	Asian	1
	South American	1

### Focus group

By the end of the course, students were invited to participate in a focus group to share in-depth feedback on their experience, and in particular to reflect on the course approach working toward transdisciplinarity. Ethical approval was obtained from the Ethical Committee of the faculty, all eight students participated in the focus group. The session lasted about 1.5 h. The focus group was led by a senior medical student (CD) who did not participate in the design or concept of the course but attended only the last sessions, who was doing an internship within the Department of Sustainable Health. During the focus group, the following two main themes were identified and addressed, on which the students elaborated extensively

- The relative contribution of the approach working toward transdisciplinarity in enriching the learning environment.
- The learning environment: experiences on the vertical (academic expert to student) and the horizontal (student to student) learning process in terms of quantity and quality of learning.

Any new important topic arisen during the focus group was considered and analyzed accordingly. The focus group was video recorded, after asking permission of all the participants, and the recording was transcribed using the free software program OtterAI (21).

### Thematic analysis

In order to analyse the content of the collective reflection captured during the focus group, a thematic analysis was used. This was conducted following Braun & Clarke's 6-step framework (22). Initial codes were generated by 3 researchers (CD, JSD, RO) independently. A combination of open and closed coding was used, some pre-set codes based on the questions asked in the focus group were used, but others were also developed and others modified as the researchers worked through the transcription. All coding was done by hand. The initial codes were then combined and grouped in themes and subthemes. In the next section, the results will be presented by theme merged in three main sections, and illustrated with relevant quotes.

## Results

**Figure 1:**
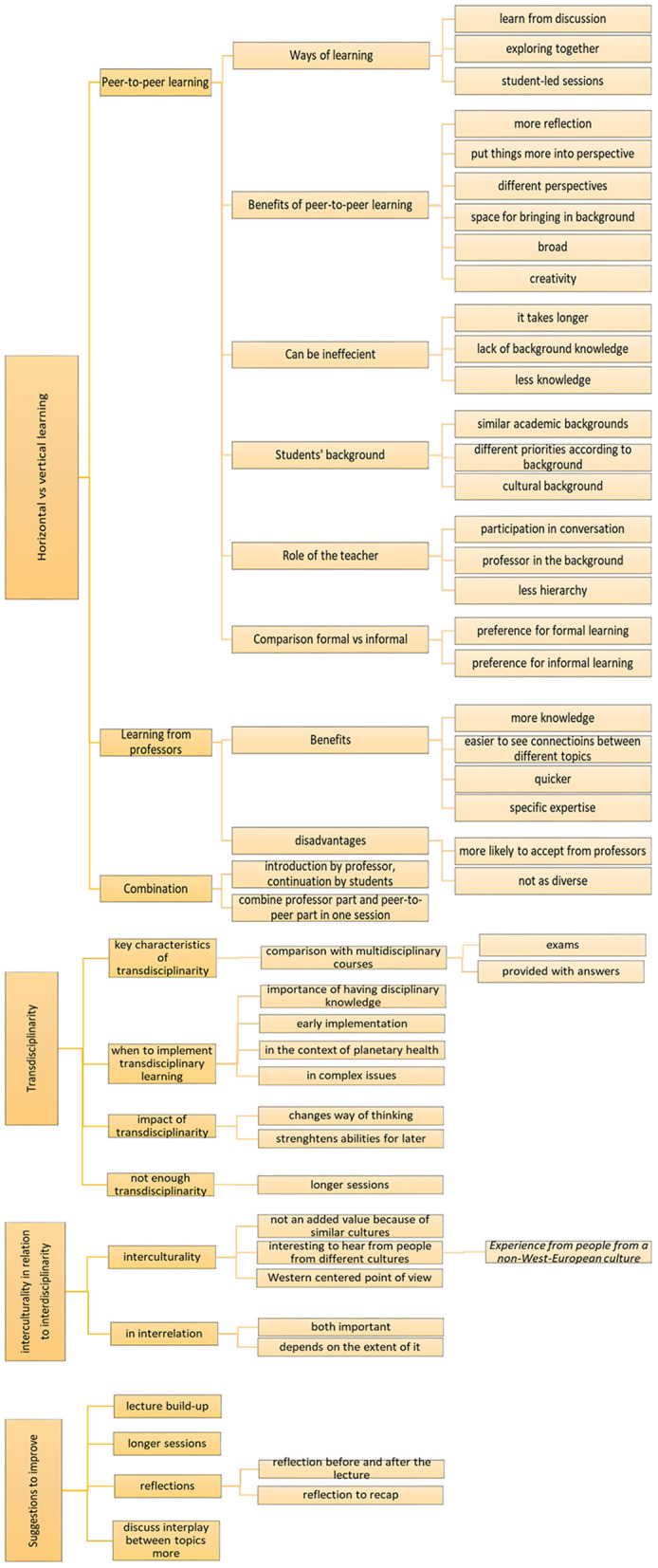
Overview of the themes, subthemes and codes used for the thematic analysis.

The focus group transcription was categorized into 4 main themes with 13 subthemes and 25 codes with 29 sub-codes (Figure 1). These were initially analyzed separately, and subsequently re-grouped into three main sections: working toward transdisciplinarity in planetary health, transculturality in relation to transdisciplinarity, and teaching and learning environment (including points for improvement). Quotes are reported without any reference to the characteristics of the responder in order to prevent their potential identification, given the limited number of participants in the focus group. Overall the students participating in the focus group produced the points of the discussion in a collaborative way, and no major disagreements worth mentioning were recorded. As such, in the results, the opinions of the participants are referred to as a unique collective source.

### Working toward transdisciplinarity in planetary health

A few important key characteristics of the learning process working toward transdisciplinarity brought up in the focus group were that it was strictly collaborative and that it implied a slower learning pace. According to the students, in this class learning was not something that could be achieved on one's own, it needed to be in a group. Moreover, it was a slower learning process compared to disciplinary learning, but it gave a broader, and more comprehensive perspective in the end. They also felt they needed to accept that the complexity of the problem posed meant that there was not always an answer to every question, differently from other courses they had attended. Overall the students considered the approach working toward transdisciplinarity not as an end goal, but as a tool that they could use on complex issues and learn with.

The students also stressed the importance of coming to the course with some disciplinary background knowledge relevant to Planetary health, which they felt only partially to have. They stressed the importance of multiple disciplinary backgrounds as contributors to increase the efficiency of learning in a context working toward transdisciplinarity. Finding the balance in the education system between disciplinary and transdisciplinary learning was felt as key.

“*I think everyone would value having an interdisciplinary approach, but then again* it *comes into my mind this collective thing of learning about each other, and you need the foundation.”*

This argument implicitly prompted a reflection on when it is appropriate to start using a transdisciplinary approach in higher education. When was the disciplinary knowledge enough to enable it? While for one student, the transdisciplinary approach came too early in their bachelor programme, causing her to feel she lacked the background knowledge for the Planetary Health course, all other students agreed that early implementation was better.

“*But I think it* [the transdisciplinary approach] *really helps our education. And … I like it and I think it's good that we implement it early on in the bachelors already.”*

All students agreed that gradually increasing the level of transdisciplinarity throughout the education trajectory could be a useful way to implement it early on but still have room for gaining the disciplinary knowledge one needs as a foundation.

Early implementation was also perceived to be good because transdisciplinary learning was felt as a way of strengthening students' abilities for later on in the education, changing their way of thinking. Students felt they gained more perspectives, started thinking more holistically, they were more open-minded, and this changed the way they dealt with problems.

“*So I think … what this approach does* [the transdisciplinary approach], *is it gets your gears turning, … it gets you thinking, it gets you analyzing, that's …, I think, the most integral part to it.”*

The specific approach working toward transdisciplinarity implemented in the Planetary health course where students from all majors were invited to participate was felt as a particular important addition to the Bachelor. It was also noted how, especially in the context of planetary health, transdisciplinarity was a valuable approach to address complex issues and learn about them.

“*For complex issues, it makes most sense to use an interdisciplinary approach because they are so … complex, they affect different areas of life. So it makes a lot of sense to use an interdisciplinary approach for complex issues.”*

The nature of a genuine transdisciplinarity in this course was questioned by the students as the backgrounds were felt not to be different enough. In addition, the students found there to be less than desirable cross-over or connection between the many topics covered.

“*We had a lot of different topics, but … sometimes it felt that they still stayed a little bit within their own side.”*

A suggested way to increase the transdisciplinarity of the course was to have longer sessions to discuss the interplay between the topics more in depth. The students noted that time-wise the discussions often got cut, and interesting thoughts were lost after the session. Pace was also deemed very important by the students as they felt it was easier to follow the train of thought when every argument was built slowly.

“*Because especially in this interdisciplinary context, I sometimes after the session, I suddenly realize, … this is a very good point, or this is a very interesting perspective, I wonder what the rest thinks about this. But then, because the next session is already on a different topic again, then that* it *gets lost.”*

### Transculturality in relation to transdisciplinarity

The students in the course did not only have a slightly different academic background, but also different cultural backgrounds. In this section, results on the relative perceived importance of both these aspects and how they influenced the learning environment is reported. The topic of transculturality was not prompted by the conductor of the focus group but came up in the discussion; it was therefore analyzed as a new topic, accordingly.

Both transculturality and transdisciplinarity were regarded as very important for the learning environment. The students acknowledged that the more backgrounds and cultures were different among the participants in a discussion, the more they learnt from these other perspectives. In this particular case, most but not all the students came from Western-European countries, so interculturality was present, but limited.

“*It was very interesting to hear from the people that were from very different areas in the world to hear what they had to add from there.”*

The students noted that transculturality was also limited in the literature list for this course; most papers concerned studies in western countries and were published in western journals. However, all students noted how it was really of added value to hear the perspectives of the students and guest lecturers that came from different areas of the world.

Importantly, some of the students raised the point that when discussing transdisciplinarity, it meant encounters with disciplines defined in a Western context. How transdisciplinarity and different disciplines are dealt with in different cultures was not included into the course. This contributed to narrowing the concept of transdisciplinarity tackling it purely from a Western hemisphere/global North perspective.

“*Interdisciplinarity, I never really realized that up until now, but of course, we think is still in the disciplines as in the westernized university structure, and we take those disciplines and then we try to link those so that the concept of how we live interdisciplinarity, or how we deal with the topic is also really, culturally based in how we structure our universities, that's true.”*

This is deemed to be particularly important for Planetary Health where the intimate relationship between humans and nature is an integral part of the picture, and where some individual and collective values and traditions and the intrinsic position of nature in human life is deeply different across cultures.

### Teaching and learning environment

Overall, the students agreed that creating this multidirectional learning environment with both horizontal and vertical learning combined was an important part of transdisciplinary teaching in the Planetary health course. When comparing peer-to-peer learning with learning from academic experts, the biggest differences according to the students were the efficiency of the learning process and the amount of knowledge students gain. Students perceived academic experts to have more knowledge and expertise and that they could bring that knowledge across in a quicker way. They could also point out connections between different points more easily.

“*… learning from a professor content wise is a bit more efficient, like you get more content in a shorter period.”*

Sometimes peer-to-peer learning felt less efficient. It is a slower journey, it took longer because peers often lacked in-depth background knowledge on topics and overall they gained less new knowledge than when learning from academic experts.

“*You're sort of lacking the basis and often you get discussions that are where we're all discussing about something we still don't really grasp.”*

“*It takes so much longer to get to a point where you have something like concrete knowledge.”*

Nonetheless, the benefits of peer-to-peer learning compared to learning from academic experts were that students felt as if they were more prone to critically reflect on what had been said, while when information comes from academic experts, they were more likely to directly accept it.

“*Learning from peers is more talking to each other, and maybe even like, disagree or, yeah, critically, critically reflect on it.”*

With peer-to-peer learning, students mostly learnt through discussions with their peers while they were exploring new topics together. Students found this a good way of learning.

“*And I learned so much in that course, just from all this, discussing.”*

Students were also exposed to multiple different perspectives together in peer-to-peer learning, which added more diversity than learning from academic experts. They noted that in more vertical settings, even when academic teachers were really conscious about the coexistence of multiple perspectives and tried to approach a topic from different angles, they were not always successful in conveying multiple points of view in a balanced way, as was the case with regards to peer-to-peer learning.

“[in peer-to-peer learning] *there's generally more diversity in perspectives and in ways to approach a topic.”*

“*Sitting there hearing from people from different backgrounds on a topic that they prepared I think I would also say that was more valuable.”*

Furthermore, the students felt there was more space to bring in creativity and their own background in peer-to-peer learning, both cultural and academic. Those backgrounds and different perspectives that every student brought in, were what made the student-led sessions so interesting to follow for other students.

“*Everyone prioritizes different things and emphasizes different things in what they want to learn.”*

According to some students, ideally, the horizontal and vertical learning would be combined in one session: with an introduction by an academic expert first, to lay the basis and gain the knowledge and have a more efficient peer-to-peer discussion after that.

“*But maybe I would even think about not having that in two separate sessions, but combining that in one session. so, you first have half an hour more lecture type by for example, the professor and then[...] have the rest of the hour a student led session, so not have the whole one and a half hours by students, but combine like the lecture by the professor with the peer to peer part a bit more.”*

The role of the teacher during this peer-to-peer discussion was more in the background, but with some interventions in the discussion. By taking part in the conversation, on the same level as the students, students noted that teachers could provide a different perspective or some more in depth knowledge, without a hierarchical structure, benefiting the overall learning environment.

“*There's not this one professor that is higher up.”*

In general having more reflection time and time to recap was suggested by some students as needed to improve the learning environment, because many topics and many points of view were discussed making it hard to remember everything and keep a balanced overview.

“*I wish we would have had a little bit more because then the knowledge can stay even more in your brain because you like recap and remember, but also you get to process it and see how other people have processed.”*

Co-teaching by more than one professor was also seen as very fruitful. When two or more experts were present in one session and the discussion developed among students and experts was seen as maximizing transdisciplinary learning, witnessing a real-life interplay between the topics.

“*Having half an hour by one expert and half an hour by the other and then have a shared discussion both with students and also active involvement of both these lecturers is really really valuable.”*

Overall, the students expressed appreciation for the course, which was evaluated well.

## Discussion

This research evaluated an innovative approach working toward transdisciplinarity teaching in the course of Planetary Health in the BSc Global Responsibility & Leadership through substantive feedback and reflections from the students collected in a focus group. Overall, having an approach working toward transdisciplinarity in the planetary health course was perceived as beneficial for the learning process of this complex field. Creating a multidirectional learning environment without a hierarchical structure improved the learning environment. Combining horizontal learning in which students learn mostly *via* discussions with peers and vertical learning in which academic experts, preferably *via* co-teaching, give students essential new knowledge needed for these discussions in a more efficient way, was key to maximizing the gaining of new knowledge and perspectives. Peer-to-peer learning and teaching was already reported as a way of improving students' critical thinking, learning autonomy, motivation, collaborative and communicative skills (13, 23); however, less evidence is available on the advantage of this technique when students from different disciplinary backgrounds are merged in one class. Importantly, students pointed out that they felt that this type of learning was possible only in a group. This implied that they regarded the experience not merely as an encounter of different disciplines as in an interdisciplinary approach, but valued the role of the interaction and cross-fertilization of disciplines, which means working toward a more substantial transdisciplinary approach.

Another important factor highlighted in the present study is that planetary health is not only a transdisciplinary field, but should also be seen as an intercultural field, with the notion that these are not two separate concepts but are complementary and interconnected. In this respect, transculturality could also promote the further evolution of transdisciplinarity by embracing disciplines beyond the traditional western approach (24). For example, spirituality in the relationship between human and environment is often not included in the complexity of planetary health studies. Nonetheless, in some indigenous communities of the Amazon forest the reality is perceived as an integrated entity of the environment, the society, the culture, the economy and the religion, without real ontological differences between them (25). Transculturality in Planetary health, therefore, not only deals with the co-existence of different cultures in the class, and often cultural differences between students origins and the place where learning takes place (26). It also needs to be accounted for in the peer-to-peer learning, team working, and problem solving typical of this discipline, and it needs to enrich the discipline content itself.

The students participating in the present research stressed the value of having a interdisciplinary and transdisciplinary courses in their university program, but also acknowledged the need for disciplinary knowledge as a foundation. Finding the right balance between enough disciplinary knowledge and early implementation of transdisciplinarity is challenging. To do so, devoting 10% of the teaching time in each discipline to transdisciplinarity was proposed in the literature, as well as having a transdisciplinary department in every university that could act as a network of all disciplines (27). Among the benefits of early implementation of transdisciplinarity there is a change in the way of thinking of the students and the teachers alike (28). Approaching problems from different perspectives, finding common language with other disciplines and more holistic thinking are skills very useful for professionals in every field, elements which are commonly not taught in conventional education (28).

Moreover, the creation of a multidirectional teaching and learning environment not only benefits the learning process but also presents an opportunity to shift (higher) education away from a top down model toward a more participatory model, where peers and lecturers can learn in partnership. A number of supervised time slots dedicated to recap previous sessions, essential learning points, and reflections was suggested by this group of students as a way to acknowledge a slower learning process improving students' ownership of the process itself.

The potential benefit of including transdisciplinary and transcultural planetary health courses in all fields of education, from professional like medicine, engineering, or law, to all natural and social science as well as liberal arts, is evident (29). At present, it is particularly striking that the subject of planetary health is barely included in medical curricula, public health curricula, or other health professionals' education. Research shows that internationally, only 15% of medical schools worldwide have incorporated climate change and health in their curriculum (30). While medical students and student associations are advocating for integration of planetary health in the curricula, medical education is slow to respond. Medical students would gain vital clinical skills by appreciating the interconnectedness of human health and environment, but also start to think more critically about the healthcare systems they work in (31).

Ideally all educational programmes would have combined courses to engage in a truly transdisciplinary approach, breaking with the disciplinary structure of the universities. This would contribute to training the next generation of leaders with increased sensitivity to environmental matters, and their complexity.

A further degree of complexity which is intrinsic to some definition of transdisciplinarity also includes collaboration with different stakeholders outside the scientific field who contribute to the construction of knowledge and the co-creation of practical solutions to social problems (16, 17). In order to achieve this, it is important to introduce the shift toward a transdisciplinary approach to science starting from the early education years. In this way, students would be well-equipped to collaborate across fields to produce knowledge and innovation with social relevance (32, 33). This can represent a further step to promote transdisciplinarity in Planetary health with a concrete and task-oriented approach.

### Strengths and limitations of the study

The main strength of the present study is that it is based on an innovative teaching method, heavily relying on an approach working toward transdisciplinary, intercultural and peer-to-peer learning, that has not yet been described in the literature. In addition, a characteristic particular to the study was that it was conducted within a bachelor programme primarily constituted out of people who identify as women in a field such as leadership, commonly dominated by men. This can be seen both as negative, due to lack of equal representation, and as positive, since it provides a unique perspective. The variety of the teacher's academic backgrounds on the other hans was an important strength of this study, as this really fostered an inter- and trans-disciplinary thinking.

The limited number of participants in the study undoubtedly constitutes a limitation. With only eight students in the course and participating into the focus group, it is hard to draw major conclusions or generalize our findings. Further, we only had one focus group and no individual follow-up interviews. This could influence the findings, as answers in groups can be influenced by others. In addition, the course leader (VG) attended the focus group as an observer, this might have inhibited some overt criticism of the course and some of the most critical reflections. It is worth noticing, though, that no further point was raised in relation to the course in the anonymous evaluation of the students which was due after the focus group. Collecting reflections through focus groups might imply some reciprocal influence on the content of the reflection, limiting the opportunity for original thought among participants. While this is true, it was interesting to hear different voices also on themes raised by the students themselves. Another important aspect is that the horizontal learning in this course was not truly built on different disciplinary backgrounds as students came from different majors yet with similar foundation education, and a pilot course we sis not yet manage to include an element of stakeholder involvement which is often associated with transdisciplinary learning. Therefore, the unique disciplinary knowledge every student had was limited and voices from outside of academia were not included, which is why it could be argued that our approach was only “quasi-transdisciplinary.” Given these limitations, it is very difficult to generalize these results. Instead, they should serve as a guide to prompt further innovative teaching ideas and to prompt additional reflection on adopting transdisciplinary approaches in the teaching and learning of planetary health.

### Conclusion

In conclusion, this study supported the notion of incorporating transdisciplinary and transcultural teaching of planetary health into undergraduate programmes as an added value, even when the primary focus is not on public health. The peer-to-peer horizontal learning opportunities within the module were seen as a way for taking advantage of the collaborative, informal teaching and community building serving the overall scope of the course. Moving beyond a pilot as the one we have described, future steps would be to incorporate external stakeholders in the educational environment in order to not only work toward but fully apply transdisciplinary teaching and learning in planetary health.

## Data availability statement

The transcript of the full data supporting the conclusions can be made available by the authors, upon request. The original recording cannot be shared to protect the privacy of participants.

## Ethics statement

The study was approved by the Ethical Committee of Campus Fryslân. All participants signed an informed consent before taking part. All participants were given the final copy of the paper before submission and given the opportunity to object the content of the paper.

## Author contributions

VG and CD: study concept and design. CD, JS, and RO: analysis and interpretation of data. CD and JS: drafting of the manuscript. CD, VG, and RO: data collection. VG, DM, and IZ: critical revision of the manuscript for important intellectual content. All authors contributed to the article and approved the submitted version.

## Funding

The study was made possible by an Erasmus + fellowship awarded to CD.

## Conflict of interest

The authors declare that the research was conducted in the absence of any commercial or financial relationships that could be construed as a potential conflict of interest.

## Publisher's note

All claims expressed in this article are solely those of the authors and do not necessarily represent those of their affiliated organizations, or those of the publisher, the editors and the reviewers. Any product that may be evaluated in this article, or claim that may be made by its manufacturer, is not guaranteed or endorsed by the publisher.
